# A Novel Approach for the Creation of Electrically Controlled LC:PDMS Microstructures

**DOI:** 10.3390/s22114037

**Published:** 2022-05-26

**Authors:** Katarzyna A. Rutkowska, Piotr Sobotka, Monika Grom, Szymon Baczyński, Marcin Juchniewicz, Kasper Marchlewicz, Artur Dybko

**Affiliations:** 1 Faculty of Physics, Warsaw University of Technology, Koszykowa 75, 00-662 Warsaw, Poland; piotr.sobotka@pw.edu.pl (P.S.); monika.grom.stud@pw.edu.pl (M.G.); szymon.baczynski.dokt@pw.edu.pl (S.B.); 2 Centre for Advanced Materials and Technologies (CEZAMAT), Warsaw University of Technology, Poleczki 19, 02-822 Warsaw, Poland; marcin.juchniewicz@pw.edu.pl; 3 Faculty of Chemistry, Warsaw University of Technology, Noakowskiego 3, 00-664 Warsaw, Poland; kasper.marchlewicz@pw.edu.pl (K.M.); artur.dybko@pw.edu.pl (A.D.)

**Keywords:** optofluidics, microfluidics, liquid crystal devices, PDMS, LC:PDMS

## Abstract

This work presents research on unique optofluidic systems in the form of air channels fabricated in PDMS and infiltrated with liquid crystalline material. The proposed LC:PDMS structures represent an innovative solution due to the use of microchannel electrodes filled with a liquid metal alloy. The latter allows for the easy and dynamic reconfiguration of the system and eliminates technological issues experienced by other research groups. The paper discusses the design, fabrication, and testing methods for tunable LC:PDMS structures. Particular emphasis was placed on determining their properties after applying an external electric field, depending on the geometrical parameters of the system. The conclusions of the performed investigations may contribute to the definition of guidelines for both LC:PDMS devices and a new class of potential sensing elements utilizing polymers and liquid crystals in their structures.

## 1. Introduction

Polydimethylsiloxane (PDMS) is a high-performance material belonging to polymeric organic silicones. The most common application of this elastomer is in microfluidics, with particular attention devoted to lab-on-a-chip and sensing devices [1,2]. Operating with negligible fluid volumes, while simultaneously allowing for the multiplication of measurement channels, a single chip of this type can replace massive and costly laboratory tools used for diagnostics, chemical analysis, or synthesis [3,4,5]. The physical and chemical properties of PDMS allow for a low-cost and reliable fabrication technology based on soft lithographic molding and embossing [6,7]. In fact, one of the most important features that determine the widespread implementation of PDMS is its relatively inexpensive and straightforward processing. As a result, designs of practically any shape, including high detail patterns (with an accuracy down to single micrometers), are achievable. Such an approach, however, requires a high-quality master mold. The latter is essential for optofluidic applications where various types of microchannels with complex geometries are needed. Additionally, the exploitation of PDMS materials allows for rapid prototyping, making new concepts easier to test. For instance, obtaining analogous photonic structures in semiconductors in such a short time period would not be possible. On the other hand, the fabrication of optical systems using polymers is generally significantly cheaper and less complicated than their manufacturing in silicon or glass [6]. Since PDMS exhibits favorable properties in terms of optical applications, it is commonly employed in optofluidic systems [1,8,9]. Specifically, it is characterized by high transmittance in a wide spectral range (from 240 to 1100 nm), including UV, which is a rare feature for polymers. PDMS exhibits negligible birefringence, and its refractive index is about 1.41 (for a wavelength of 589 nm) [10]. Moreover, the properties and processing of PDMS may be relatively easily tailored and modified by adding catalysts and fillers [11,12].

The LC:PDMS specific structures explored in this research work are optofluidic systems in the form of microchannels fabricated in PDMS and filled with liquid crystalline material. The proposed approach represents a relatively new field of studies, and therefore LC:PDMS waveguiding systems are still limitedly reported on in the literature [13,14,15,16,17]. One of the factors contributing to their uniqueness is the application of LCs as an active element of the system. It distinguishes the described structures from typical optofluidic devices in which isotropic liquids are commonly used [1]. It means that the proposed solution may gain advantages related to the characteristic features of LCs, such as a significant anisotropy of their physical properties and high sensitivity to external fields and factors, combined with low cost and low energy consumption at the same time [18]. Such features allow for easy tunability and reconfiguration. Indeed, LC:PDMS photonic systems seem to be perfect candidates for sensing applications while offering electrically, magnetically, and optically controlled cores of easily switchable and reconfigurable waveguides. With a wide range of liquid crystal compounds available, selecting the most suitable one for specific applications is just a formality. This statement is particularly true in relation to our up-to-date experience and experimental observations indicating no chemical interactions between PDMS and typical liquid crystalline materials [19]. Equally important is the fact that besides the possibility of complex microstructures fabrication, PDMS may be used to design and develop a substrate with the desirable anchoring energy for LC molecules. It allows adjusting the LC pretilt angle from 0 to 90 degrees as a function of the PDMS content in poly(vinyl cinnamate) with an azimuthal anchoring energy of ~6 × 10^−6^ J/m^2^ at intermediate pretilt angles [20]. PDMS thin films have also been used as alignment layers for LCs in display applications with a possible linkage between PDMS curing conditions (i.e., time and temperature) and the final surface free energy value (in the range of ~20–30 × 10^−3^ J/m^2^) [21,22]. With such a low surface free energy (lower than that for commercial polyimide SE-4811 used in the display industry) within a wide temperature range (up to 80 °C), the PDMS films are capable of promoting the vertical alignment (VA) of liquid crystal molecules [22]. Eventually, untreated PDMS is characterized by high hydrophobicity and low surface anchoring energy, allowing for the spontaneous homeotropic (i.e., vertical) orientation of LC molecules on the elastomer surface [19]. It has to be underlined that such considerations related to the ordering of liquid crystal molecules inside photonic microstructures are not without reason in the present case. It should be remembered that the functionality of LC:PDMS structures, especially in relation to optical applications, highly depend on the LC molecular arrangement. The essential condition for receiving and controlling the anisotropic properties of LC material is to obtain a specific orientation of its molecules within the sample volume. Typically, the surface ordering methods are employed by applying suitable substances, e.g., rubbed polymer films (generally polyimides) for planar and surfactants (such as lecithin or chromolane) for homeotropic (vertical) orientation, respectively [23,24,25]. Alternative technologies for LC alignment [26] include the deposition of obliquely evaporated thin films (oxides), micro-grooving, nanopatterning, as well as a non-contact method of the photoalignment [27]. Whichever technique is applied, strong anchoring is obtained for the azimuthal anchoring energy of more than 10^−5^ J/m^2^. The latter may be achieved in the rubbed polyimide layer (~10^−4^ J/m^2^) [24,25] or in the photo-aligning materials (up to 7.2 × 10^−5^ J/m^2^ for sulfuric azo-dye SD1) [27,28] but with the assistance of specialized equipment and after suitable sample preparation. Therefore, it is not surprising that structures made of PDMS attract considerable interest as this material allows, in principle, for the intrinsic vertical orientation of liquid crystal molecules (with no either orienting layers or additional processes required) [19,21,29] with an azimuthal anchoring energy [20] of about one order of magnitude lower than for lecithin [30].

Such an orientation of LC molecules can be relatively easily changed and controlled by applying an appropriately formed electric field to LC:PDMS structures, thus imparting additional flexibility to microfluidic waveguiding systems. Unsurprisingly, an essential step in working on described optofluidic microdevices is designing and creating an adequate electrode arrangement. By applying suitable voltage to the electrodes of a specific shape and configuration, it should be possible to change the LC optical parameters, e.g., to guide the light beams desirably in optofluidic systems. Additionally, developing electrodes of a particular type (e.g., periodic) may introduce novel tuning and sensing options. Such manipulation in the electrodes’ shape is highly desirable and opens up new possibilities for an even more sophisticated development of microfluidic devices.

The literature shows that various possible electrode configurations could be achieved in PDMS systems. Most of them are limited by technological constraints related to difficulties in forming conductive layers on elastomer surfaces because of PDMS’s low adhesion and low surface energy [7]. Another major bottleneck is the precise microelectrodes patterning being affected by PDMS elasticity, especially in the case of deep microchannels. Therefore, it is evident that many technological features have to be considered to create a functional electrode system of good quality, or novel solutions should still be searched for. When analyzing possible methods to achieve conductive structures on the PDMS surface or into the bulk, bonding with metals or metallic oxidates and the addition of metallic nanoparticles into the PDMS volume [13] may be potentially explored. In many cases, chemically inert materials (e.g., platinum) are preferred, which unlike chemically active ones (e.g., gold), do not react with the sample and thus do not introduce additional drawbacks, such as blocking the flow inside the channels. Among the different options for electrode formation in PDMS structures, coating with a layer of a transparent conducting polymer (PEDOT:PSS) [31,32] or the deposition of thin metal layers (titanium, nickel, chromium, and gold) on the PDMS surface [33] may be distinguished. Unfortunately, metal layers cannot be firmly attached to PDMS, making it problematic to bond those two materials together. Therefore, using additional intermediate layers to facilitate and optimize the electrode deposition process may be beneficial. For instance, platinum electrodes exhibit good adhesion to the substrate by using a thin titanium layer between the platinum and the elastomer [34]. The deposition of metallic layers may also be carried out after PDMS surface activation in oxygen plasma (e.g., in the electroplating process with gold [16]) [35]. The surface modification improved the adhesion and abrasion resistance of conductive components manufactured on PDMS when using airbrushing as a low-cost alternative for fabricating flexible electronics [36]. Another commonly investigated approach involves sputtering different conducting materials directly onto the elastomer surface [37,38,39]. However, even when adjusting the temperature and duration of the sputtering process for ITO (indium tin oxide) to minimize the negative effect of the cracking and wrinkling of the PDMS surface, this technique leads, in general, to structural damage [40]. Despite all the drawbacks, attempts to apply metallic electrodes to PDMS are described in a significant number of scientific papers with selected fabrication trials and electrode applications, including semi-transparent Ag electrodes deposited on pre-strained PDMS via the DC magnetron sputtering system suitable for stretchable electronics [41], a high-sensitivity, a stretchable thin-film sensor for wearable devices based on the Ag nanowire network deposited on the PDMS surface with the use of capillary force lithography [42], a flexible three-electrodes electrochemical sensing device consisting of Au and Ag electrodes fabricated with the use of chemical plating and electrochemical etching [43,44], a conductive homogeneous gold layer on native PDMS obtained by chemical plating and electrochemical etching developed as a component for renewable electrodes and complex integrated biochips [44], copper electrodes covered with a thin PDMS layer in the spin coating process applied in wearable applications without the necessity of using a gel as the electrode-skin interface [45], metallic nanostructures obtained on the PDMS surface using holographic nanopatterns of a photoresist layer as structure template [46], and microelectrodes obtained with the direct inkjet printing of gold and silver nanoparticles on PDMS for functionalized microfluidic systems (including electrochemical sensors) characterized by good compactness, continuity, and conductivity [47,48]. Unfortunately, none of the processes and methods described above may be considered as universal, well-developed, easy in application, and providing satisfactory conditions at the same time. For this reason, alternative and more reliable methods were sought. Another class of possible arrangements is based on using metallic particle-PDMS-based conducting composites [49,50,51], allowing for two- and three-dimensional electric signal connectors to be constructed and easily bonded or embedded into PDMS microfluidic chips (with good sealing between carbon electrodes and PDMS substrates achieved by using oxygen plasma treatment). To make this possible, adequate synthesis processes have been developed to transform pure PDMS materials into conductive ones by adding, e.g., carbon black powder [52,53,54] or by combining it with silver [50] or nickel [55] particles, at a suitable concentration. However, to exhibit conductive properties, a high number of carbon nanoparticles are required [50,54]. As the concentration of the dopant particles increases, the changes in the mechanical characteristics of PDMS composites become evident [52], with materials becoming stiff, difficult to process, and easy to break. Un surprisingly, the studies on electrodes manufacturing were directed towards different technologies, namely the use of liquid metals [56,57,58,59,60,61], which greatly shortened the fabrication time and lowered the cost compared with traditional deposition or sputtering techniques. Particular attention was devoted to gallium (Ga) and its liquid (at room temperature) alloys which are attractive alternatives to toxic mercury. Specifically, Ga-based liquid metals may be injected [61,62,63], imprinted/patterned [60], or 3D printed [58,59] on the PDMS substrate, promoting rapid and superficial processing. Room temperature direct-writing and 3D printing facilitate the rapid fabrication of complex geometries with dimensions of as small as 10 μm [58] and a minimum line width of about 2 μm [59]. Maintenance of the same level of precision in microelectrodes fabrication, but without the necessity for additional equipment, is made possible when injecting a low-melting-point alloy (such as eutectic gallium indium) into microchannels. The latter strategy helps to pattern and encapsulate highly reconfigurable electrodes, which may be applied in integrated microfluidic systems. Importantly, the channels defining the shape and position of the microelectrodes may be fabricated simultaneously with other microfluidic channels (avoiding the multi-step fabrication procedure), being inherently aligned with and moreover, placed close to (even in the direct contact) the microfluidic channel [61]. The only limitation may be the filling of complex structures with liquid metal, but the utilization of a vacuum in the case of dense and branched microelectrodes (with features as small as several microns) with liquids was suggested [64]. Another method is to use a liquid with a lower density, e.g., conductive ink [65]. Reliable and straightforward saltwater electrodes were also demonstrated, performing the same operations in microfluidic devices at similar voltages despite their relatively low conductivity [66].

Concluding the introductory part, it has to be underlined that this work aims to design and investigate a novel class of optofluidic LC:PDMS systems with particular emphasis on determining their performance in applying an external electric field. It demanded supplementing typical microfluidic devices with new elements such as anisotropic liquid crystals and electrodes in the form of microchannels filled with a liquid metal alloy. Such an approach provides an innovative solution that can advance the knowledge on the practical application of polymers in photonic devices, particularly in waveguiding and sensing systems. It is worth noting that the technology used so far to obtain electrically driven LC:PDMS structures with flat electrode systems sputtered on the polymer surface is complicated and may additionally cause damage to the microstructures themselves [14]. The primary motivation behind the proposed research topic was thus to develop an alternative way of making electrodes in the form of a microchannel and demonstrate that such a method can be successfully used to tune the discussed optical microsystems electrically. Specifically, it was proven that employing microchannels filled with liquid metal alloys can produce electrodes of any shape and geometry and, above all, place them near the liquid crystal channel. Such a configuration allows for the simple and efficient control of the propagation properties of LC:PDMS devices with promising applications for highly integrated optofluidic systems.

## 2. Materials and Methods

### 2.1. Numerical Modelling Methods

**Figure 1:**
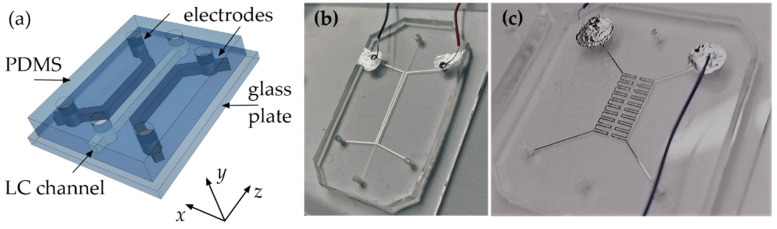
(**a**) Schematic representation of the LC:PDMS structure on the glass substrate. (**b**,**c**) Fabricated samples with side channels filled with a eutectic gallium-indium alloy.

The first stage of the study was to design the structures using the AutoCAD software (Autodesk, Dublin, Ireland) while considering the technological capabilities. At the same time, suitable diversity of the proposed configurations was ensured to check how the different geometries would affect the experimental results. Particular attention was paid to analyzing various electrode configurations. The validity of the proposed shapes was verified based on the numerical calculations performed. The latter allowed for the determination of the spatial distribution of the electric field intensity depending on the system architecture and, as a result, the selection of the optimal geometrical and physical parameters of LC:PDMS microstructures for fabrication. The numerical calculations were performed with the COMSOL Multiphysics simulation package (COMSOL, Inc., Burlington, USA), commonly applied to model various physical problems by solving appropriate partial differential equations using the finite element method. Depending on the specificity of the problem, the calculations may be performed in one, two, or three dimensions [67], preceded by the inherent preliminary steps of mapping the actual experimental conditions (such as the system geometry, material properties, and boundary settings) and generating an appropriate computational mesh. The electromagnetics-electrostatic (AC/DC) computational module in 3D was specifically used in this work to determine the spatial distribution of the electric field intensity. Because of the variety and complexity of the electrodes’ configurations planned for the LC:PDMS structures, their geometries were designed in the AutoCAD software as a two-dimensional drawing and then converted into a three-dimensional structure by providing the appropriate height. The latter size was set at 30 μm, which is close to the actual height of the microchannels intended for use in the first tests. Models of individual structures consisted of two channel electrodes and a central channel placed between them (see, e.g., Figure 1). For the purpose of numerical calculations, those microchannels were additionally placed in a cuboid (with the gaps corresponding to the shape of the channels), forming the surrounding structure (made of PDMS) and thus establishing the range of the calculation volume. An important step before starting the calculations was to assign the material parameters to the individual parts of the structure to be numerically modeled. For the specific type of simulations to be performed, each material’s electric permittivity (ε) had to be determined. The average value of 9.9 was assumed for the liquid crystalline material (injected into the central channel). It was calculates as ε_avr_ = (2ε_⊥_ + ε_‖_)/3, where ε_⊥_ = 5.21 and ε_‖_ = 19.28 are the electric permittivity at kHz frequency in the direction perpendicular and parallel to the molecular long axis for E7 nematic liquid crystal, respectively [68]. A dielectric constant value of 3.3 corresponding to the eutectic gallium-indium alloy [69] was assigned to the pair of side electrode channels, while a permittivity of 3.1 was fixed within the cuboid representing a PDMS elastomer [70]. Once the material parameters were established, appropriate boundary conditions were considered. For this purpose, electrical potential values (assumed to be 0.5 V and −0.5 V for the first and the second electrode, respectively) were assigned to obtain a potential difference of 1 V. Moreover, a continuity boundary condition was specified at the interface between the LC channel and PDMS, and zero charge/symmetry conditions were set at the PDMS external walls, which are the default settings at the outer boundaries of the model. Subsequently, the considered volume was divided into smaller parts following the assumptions of the finite element method used by the software. The generated computational mesh was densified in the area of the central channel, for which the determination of the electric field intensity spatial distribution is of particular importance in terms of the analyzed problem (while giving the conditions for an efficient change in LC molecular orientation under applied voltage, thus affecting the optical properties of the system). Please note that throughout this paper, any reference to the electric field intensity exclusively pertains to its *x*-component.

### 2.2. Fabrication of LC:PDMS Microstructures

Due to the simplicity of PDMS processing, it can be used for the large-scale production of microfluidic systems and single structure fabrication. The basic and the most common manufacturing method is the cast-molding technique, where a flat plate with a convex pattern (i.e., the mold), which is the negative of the channels, is applied. The utility of the final PDMS microstructure strongly depends on the mold quality. The latter may be fabricated using various technologies, differing in the complexity of the resulting structure, the duration of the manufacturing process, and most importantly, the price. Other important issues to consider are the specialized equipment requirement and the inability to use the mold repeatedly without damage. The low-cost methods for the fabrication of the mold described in our previous papers [19,71], including UV-curing of the capillary film, milling in PMMA, and 3D printing in the polymer resins using SLA technology, were found to be of insufficient quality for the needs of the complex periodic structures considered in this work.

A multi-step manufacturing procedure was used to fabricate the desired structures (as exemplified in Figure 1b,c) by transferring the designed pattern of microchannels through various complex technological processes, with each of them being discussed rather concisely in this paper. All details and exact technological parameters are described very thoroughly in [19]. The applied approach includes the fabrication of a photomask using the electron beam lithography and the SU-8 photolithography processes on the silicon wafers, respectively, compressing structures with a high aspect ratio and dimensions down to single micrometers. Actual optofluidic systems were obtained by pouring a liquid PDMS into the mold and then bonding the crosslinked casting (containing a required pattern embossed on one of its surfaces) with a glass substrate. In this regard, the temporal surface activation by exposure to oxygen plasma was necessary, thereby facilitating a permanent connection and durable sealing of the channel structures. The thickness of the device itself was of about single centimeters (which is mainly the thickness of the PDMS layer bonded to the standard 1-mm-thick microscopic glass). Each fabricated sample contained a set of holes allowing for the liquid crystal and the electrode material to be introduced into the relevant microchannels. These vertical inlets must be drilled on both ends of the microchannels to allow air to escape during the filling of the structures. Their shapes and sizes are irrelevant and related to the technique of drilling them. The PDMS was cooled below its glass transition temperature (about −123 °C [72]) using liquid nitrogen to increase drilling precision. The wide-range thermal stability of PDMS makes repeated cooling and heating unlikely to damage the material. Despite the flexibility of the PDMS material, the assumed rectangular cross-sectional shape of the central and side channels was perfectly reproduced in the elastomer volume. High-resolution photos of the channels (performed with SEM) are presented in [73].

Before testing the samples, the central channel of the structure was filled with an E7 nematic liquid crystal (NLC) mixture (provided by Merck, Darmstadt, Germany). This particular liquid crystalline material was selected due to previous observations indicating the relatively rapid formation of the homeotropic alignment on the PDMS surfaces in contrast to the other NLC materials tested [19]. To ease the filling process, it was convenient to heat the substance above the nematic-to-isotropic phase transition temperature (i.e., above the clearing temperature *T_NI_* which is about 58 °C for E7 NLC [74]). Then, a small dose of the liquid crystalline material was combined with the 0.4 mm syringe needle into the microchannel in PDMS. It was possible to fill the entire length of the microchannel simply by the action of the capillary forces (when ensured that a pair of inlets were drilled adequately at the ends of the channel). Eventually, transition to the nematic phase occurred spontaneously at room temperature. The dynamic of channels infiltration with NLC material and the temporal stabilization of the molecular arrangement in different PDMS microstructures are described in [19]. It has to be underlined that all tests related to the analysis of the LC molecular (re)orientation were performed in stationary conditions—i.e., with no additional forces (such as stretch or pressure) applied to the sample.

One of the novelties introduced in this work is based on employing the LC:PDMS structures with electrodes in the form of microchannels filled with conductive fluid. The main advantage of the proposed solution is the possibility of placing the electrodes in the direct vicinity of the central channel (as shown for example in Figure 1), allowing thus for the dynamic and straightforward control of the optical properties of the liquid crystalline waveguides. Moreover, the proposed approach makes it possible to easily obtain the electrodes of arbitrary geometries and complex (e.g., periodic) shapes with very high precision without the need for other processes (e.g., introducing intermediate layers). Microchannels filled with an electrically conductive gallium-indium-based alloy seemed to be perfect candidates to solve all issues (such as brittleness, deformations, delamination, or technological complexity) mentioned in the introduction of this paper and related to conventional solid electrodes fabrication methods. Importantly, unlike other typical metal liquids, which are often highly toxic at room temperature, those based on GaIn are safe and proven to be non-reactive with PDMS materials. Eventually, the high-purity eutectic gallium-indium alloy (EGaIn, provided by Sigma-Aldrich Chemie GmbH, Taufkirchen, Germany), containing 75.5% gallium and 24.5% indium by weight, was chosen. Its relatively low viscosity (2.0 × 10^−3^ Pa·s) and high electrical conductivity (3.4 × 10^6^ S·m^−1^) [75], together with its rheological properties at room temperature, allowed for the formation of metal electrodes within the microchannels of suitable cross-sectional dimensions. The side channels acting as the electrodes were filled with EGaIn alloy using a specially cut and sharpened needle. Since it was challenging to introduce the eutectic inside the channel in some cases (i.e., for narrow channels), it was necessary to additionally introduce it using another syringe through an opening located at the opposite end to the location of substance introduction. This action ensured that the EGaIn alloy effectively filled the entire microchannel forming the electrode. Figure 1b,c show the photos of the selected samples fabricated by introducing eutectic into the investigated PDMS structure.

It should be mentioned here that during the laboratory tests, it emerged that the reduction of the microchannels’ cross-sectional dimensions (which is essential when considering the use of the designed structures as the waveguides for visible and near-infrared light) made it impossible to introduce the EGaIn alloy inside the microchannels (despite all supporting actions described above). For this reason, we decided to use a typical electrolyte, i.e., a 3 mol/L potassium chloride solution (Sigma-Aldrich Chemie GmbH, Taufkirchen, Germany) with a much lower viscosity (1.012 × 10^−3^ Pa·s) [76] and density (1.12 g/mL) [77] when compared to EGaIn. The electrical conductivity of the aqueous KCl solutions for different concentrations (in the range 0.03–4.0 M) and temperatures from −8 °C to 25 °C may be found in [78], with a value of about 1 × 10^4^ S m^−1^ for 3M KCl at room temperature. The performed investigations revealed a similar efficiency and comparable values of the steering voltages for both types of electrodes, as described in Section 3.2.

### 2.3. Testing the Influence of an Electric Field on LC Molecular Orientation

**Figure 2:**
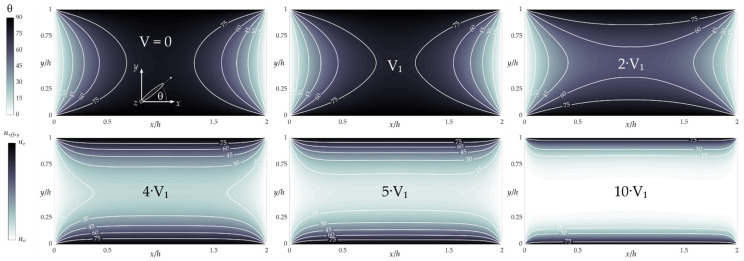
Results of numerical simulations showing molecular reorientation under the influence of the electric field (with the assumption of the flat electrodes located along the vertical sidewalls—i.e., only the *x*-component of the electric field and thus the reorientation taking place in the *y*-*x* plane is considered). Effective refractive index for *y*-polarized light beam (see the color bar in the bottom-left corner) is calculated as: *n_eff,y_* = *n_o_n_e_* (*n_o_*^2^cos^2^θ + *n_e_*^2^sin^2^θ)^−1/2^. For an E7 NLC V_1_ = 1 V (when taking ε_⊥_ = 5.21, ε_‖_ = 19.28 as electric permittivity at 1 kHz, and K = 17.1 × 10^−12^ N as the elastic constant [68]). Ordinary and extraordinary refractive indices of E7 NLC are equal to *n_o_* = 1.522 and *n_e_* = 1.739 @ 589nm, respectively [74].

As already mentioned, liquid crystals are characterized by the significant anisotropy of most physical parameters. Regarding optical properties, a typical nematic liquid crystal such as E7 is a uniaxial birefringent medium with the direction of the optical axis coinciding with an average direction of the long molecular axes arrangement. Therefore, the practical use of LC:PDMS structures as photonic devices is possible only when the orientation of LC molecules is fully identified and controlled. By assuming the presence of the stiff homeotropic boundary conditions on PDMS surfaces, it is possible to numerically determine (by solving the corresponding Euler–Lagrange equations to minimize the system’s free energy) the molecular arrangement in the LC channel cross-section [14], as shown in Figure 2. One of the efficient methods for changing the orientation of liquid crystal molecules (and thus the effective refractive index within the NLC layer, see the bottom-left side of Figure 2) is the application of an external electric field. In this case, the molecules were reoriented, resulting in an arrangement for system free-energy minimization [18,23,24]. For an E7 NLC, characterized by positive electric anisotropy, the molecules tended to align with the electric field direction when the voltage was applied, as shown in Figure 2. The latter presents the results of numerical simulations performed according to the methodology described in [14,79,80].

In relation to the topic of electrical conductivity, it is worth noting that despite the careful purification of the liquid crystalline material, there may remain a residual concentration of ionic impurities, leading to a considerable ionic electrical conductivity, σ ≈ 10^−7^–10^−12^ Ω^−1^ cm^−1^ (with the ions forming near the electrodes) [25]. An alternating electric field was applied to avoid possible electrohydrodynamic instabilities in the nematic (related, e.g., to the nonuniform distribution of the space charge concentrated near one of the electrodes). In addition, special efforts to avoid the electrolyte entering the central channel with the liquid crystal material were made during the filling of the structure.

In the performed experiments, the RIGOL DG4062 generator (RIGOL Technologies EU GmbH, Gilching, Germany) and the A800DI amplifier from FLC Electronics (Partille, Sweden, allowing a 200-fold amplification of the generated electrical signal) were used to apply the voltage of a specific value. A pair of electrodes was connected to the amplifier, and the LC molecules in the central channel of the LC:PDMS structure were reoriented using a sinusoidal electrical signal at 1 kHz. The observations were performed for different values of an electric voltage applied to the sample, and peak-to-peak values (*V_pp_*) are presented in Section 3.2 describing the results of the experimental tests.

A digital microscope (KEYENCE VHX 5000 with a universal zoom lens VH-Z20 of 20–200× magnification, Keyence International, Mechelen, Belgium) was used to observe the effect of the electric field on the NLC molecular orientation. Standard polarizing optical microscope (POM) measurements were conducted with white light transmitted (along *y*-direction according to the notation used in, e.g., Figure 1a and Figure 2a) through the sample placed between two polarizers. The axes of the latter (crossed in most cases) are schematically marked by relevant symbols included in photos from the experiments (presented in Section 3.2). In most cases, samples were positioned so that the axis of the liquid crystal channels was aligned at an angle of 45 degrees to the polarizer axis.

## 3. Results

### 3.1. Results of Numerical Simulations

Before starting the actual experimental work, a system of electrodes for applying an external electric field to the samples had to be designed. Different shapes of the electrodes were considered, and their suitability was verified by performing numerical calculations to determine the spatial distribution of the electric field intensity depending on the geometry of the electrodes. It allowed for the adoption of specific geometrical and physical parameters of the designed LC:PDMS structures, consequently selecting their optimal characteristics.

**Figure 3:**
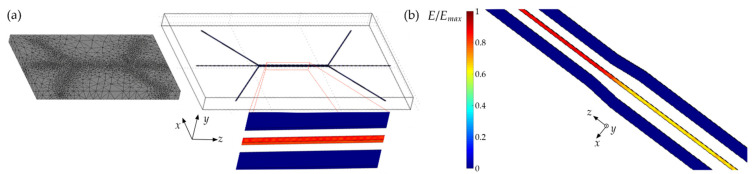
(**a**) Exemplary geometry of the LC:PDMS sample (consisting of two straight channel electrodes and a central LC channel placed between them) analyzed in the COMSOL Multiphysics simulation package. The computational mesh was densified in the channels’ region (see the scheme on the left). Normalized electric field intensity distribution in the central channel is essential for further analyses related to the imposed molecular reorientation (and effective refractive index distribution). (**b**) The normalized electric field in the LC channel located between the electrodes gradually approaching each other. Please note that the values of electric field intensities beyond the channels are omitted for the clarity of presentation.

After performing the numerical simulations according to the methods and assumptions described in Section 2.1, the achieved results were presented in graphical form as multicolored areas indicating the different values of the electric field intensities. The results obtained for an exemplary structure of relatively simple geometry (i.e., straight side electrodes) are shown in Figure 3a. Please note that for a better representation of the spatial electric field intensity (precisely speaking, normalized *x*-component) distribution within the microchannel, the visibility of the surrounding cuboid (made of PDMS) was disabled in the software when plotting the results. Consequently, white color was used in this region, providing no information about the electric field intensity. For this reason, the results shown in this section refer only to the values of the normalized electric field intensity calculated within individual liquid crystalline channels and to the influence of geometrical parameters of the electrodes on the obtained results, entirely omitting information regarding the areas between them. Moreover, the corresponding color scale was normalized for each considered case by dividing the electric field intensity by its maximum value.

Moving on to the relevant part of the numerical simulations, the possibility of changing the electric field intensity in the LC waveguide area by varying the distance of the side electrodes from the center channel while keeping the electric potential difference constant was numerically tested. For this purpose, a model structure was designed in which two electrodes were gradually shifted away from the central channel by 14 μm for every 2.7 mm of length. The widths of the LC and side channels were set to 15 μm and 50 μm, respectively. The results of these simulations are shown in Figure 3b, where two example segments, corresponding to two different distances (of 38 μm and 52 μm) between the side and the central channels, are presented. Based on this, it can be concluded that by modifying the position of the electrodes (for the main channel), it is possible to control the value of the electric field intensity and thus the properties of the entire system. For this reason, a structure of such a type was selected for fabrication to verify the efficiency of molecular reorientation under the influence of an external electric field in further experimental tests.

**Figure 4:**
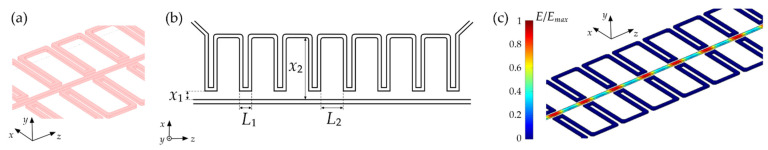
(**a**) Example of the electrodes considered for application in the LC:PDMS system to obtain the periodic modulation of the effective refractive index within the LC channel. (**b**) Geometrical parameters to be modified in the electrode design are *x*_1_—the distance of the electrode from the channel, *L*_1_—the length of the section close to the channel, *x*_2_—the distance of the electrode section further from the central channel, and *L*_2_—its length. (**c**) Normalized electric field intensity distribution obtained for *x*_1_ = 50 μm, *L*_1_ = 150 μm, *x*_2_ = 700 μm, *L*_2_ = 300 μm.

Keeping in mind that the electrically tuned LC molecular arrangement directly affects the waveguide channel’s effective refractive index, the next stage was to verify the possibility of obtaining a periodic change in this quantity in the adequately designed microstructure. Such a concept requires the precise planning of the electrodes’ geometry. Eventually, the electrodes consisting of periodically repeated sections located alternately closer and farther from the central channel were proposed, making it possible to obtain periodic changes in the electric field spatial distribution. An exemplary arrangement is shown in Figure 4a, with general geometrical parameters indicated in the schematic drawing presented in Figure 4b. Specifically, one of the periodic electrodes in the structure is visible in the scheme, while the second is considered to be flipped symmetrically with respect to the central channel. Please note the roundness of the channels in the regions where they fold at a 90-degree angle. Such a solution (i.e., with no sharp corners) eliminates the places where air bubbles may remain after filling the microchannel with conductive fluid.

For the proposed configuration of electrodes, the concept’s relevance was first verified (for widths of all channels equal to 50 μm), and then different variants of the structures with similar channel shapes were checked to determine their optimal parameters. The results of the corresponding numerical calculations are shown in Figure 4c. Accordingly, it may be concluded that it is possible to meet the assumptions adopted in the design of the structure and to achieve the spatial periodicity of the electric field intensity in the central channel. It proves that the considered geometry type can be potentially used in experimental conditions to obtain areas of different orientations of LC molecules when an electric voltage is applied to the structure.

**Figure 5:**
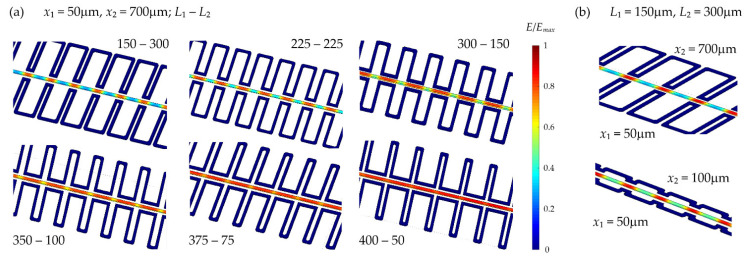
(**a**) Results of numerical simulations for the electrodes with variable duty cycles. The corresponding dimensions are given according to the *L*_1_–*L*_2_ convention, representing the length of the sections closer to and further from the central channel, respectively. The other geometrical parameters are the same for each structure, i.e., the channel width is 50 μm, and the distances between the side channel and the central channel are equal to 50 μm for the closer electrode section and 700 μm for the further one. A joint color scale was applied for the results obtained for different electrode configurations for ease of comparison. (**b**) Comparison of the results obtained in the systems with the same lengths of the periodic electrode segments (i.e., *L*_1_ and *L*_2_ are the same in both cases) but with the further section (of the length of *L*_2_ = 300 μm) moved from a distance of 700 μm to a proximity of as near as 100 μm from the central channel.

Subsequently, the geometrical parameters of the structure were modified to find the optimal ones. Firstly, the lengths of the sections located closer and further from the central channel (which are marked as *L*_1_ and *L*_2_ according to the schematic drawing in Figure 4b, respectively) were changed. The results obtained for different variants are summarized in Figure 5a.

Based on the results presented in Figure 5a, the importance of the selection of appropriate geometrical parameters for the designed LC:PDMS systems becomes apparent when it comes to the possibility of obtaining the expected spatial modulation of electric field intensity in the area of the liquid crystal channel. As the length of the electrode segment closer to the center increases (compared with the length of the part further away), the contrast in the electric field intensities becomes smaller and smaller until the field averages over the entire length of the microchannel. Such a situation occurs, for example, for *L*_1_ = 400 μm and *L*_2_ = 50 μm, for which the value of the electric field intensity is almost constant along the whole LC microchannel despite the presence of periodic electrodes. It excludes structures with too densely arranged sections of the periodic electrodes from further experimental tests because of the uniform reorientation of the LC molecules along the microchannel length in this case. In contrast, the optimal conditions (resulting in the greatest possible difference between the electric field intensities in the individual sections of the microchannel, which means the maximum modulation in effective refractive index along the propagation length) may be achieved for the periodic electrode with segment lengths of *L*_1_ = 150 μm and *L*_2_ = 300 μm. A structure with such geometrical parameters was fabricated for tests in experimental conditions. Additionally, we also decided to produce the system with parameters *x*_1_ = 300 μm and *L*_1_ = 150 μm, for which the contrast is lower, but the electric field in the central channel is still not averaged. The tests performed in such alternative structures allow for a comparison of the efficiency of the LC molecular reorientation under the influence of an external electric field in microfluidic systems with different electrode geometries. To make numerical analyses more complete, the graphs in Figure 5b compare the results obtained for the structure with *L*_1_ = 150 μm and *L*_2_ = 300 μm after reducing the distance of the electrode section furthest from the center to the value of *x*_2_ = 100 μm. It shows that bringing the more distant part of the electrode closer to the LC channels leads to a decrease in the spatial modulation of the electric field intensity.

**Figure 6:**
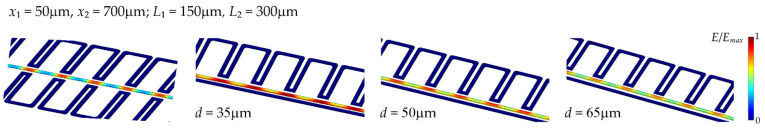
The results obtained for different electrode designs intended to be applied to achieve a periodic modulation of the effective refractive index in the LC channel. Two periodic electrodes and a system of one periodic and one straight electrode were considered, with the latter located at the variable distance *d* from the central channel. Again, a common color bar is provided for better comparison of the results.

To identify possible solutions and select the optimal electrode configuration, the system presented in Figure 4c was modified so that only one electrode was of periodic shape while the second one remained straight. Such a solution would be technologically reasonable, reducing manufacturing complexity and facilitating subsequent infiltration with an eutectic. Calculations were performed for different distances between the non-periodic electrode and the central channel. The exemplary results for double and single periodic electrode configurations are presented in Figure 6.

The main difference between the two analyzed systems with different solutions in the context of electrode geometry (shown in Figure 6) is electric field intensity being asymmetric with respect to the central channel axis for the design with a non-periodic electrode. Moreover, the spatial modulation in the electric field intensity is also unfavorably lower for the sample with the straight electrode. Even if the reduction in the axial asymmetry in the electric field distribution seems accessible by moving the non-periodical electrode aside, its increased distance (*d*) from the central channel makes the discrepancy between the extreme values of the electric field intensity even smaller. Therefore, further analyses and calculations were limited to the structures with two periodic electrodes.

### 3.2. Experimental Results

As already mentioned, the critical aspect of the performance of liquid crystal systems is the well-defined and controlled alignment of the liquid crystal molecules on the boundary surfaces and in the entire volume of the sample. Therefore, after filling the microchannels with the selected liquid crystalline material, an investigation was performed to determine how the molecules are anchored on the PDMS channel walls. The samples were tested using a polarizing microscope so that the liquid crystalline channels were positioned at 0 (90) and 45-degree angles to the crossed polarizers axes.

**Figure 7:**
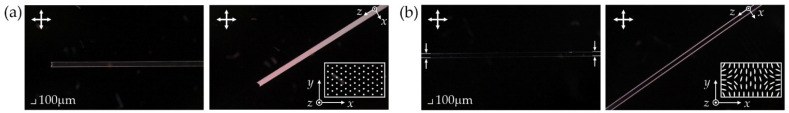
Photos taken with a polarizing microscope showing the molecular arrangement immediately after filling the central channel with liquid crystalline material (**a**) and after the homeotropic alignment on the channel sidewalls was established (**b**). The schematic representation of the LC molecular arrangement in the channel cross-section is given for each case for clarity. Additionally, in the third photo, the markers to make it easier to locate the channels that were added.

It was observed that the long axes of the LC molecules were oriented initially along the channel axis without being affected by the limiting PDMS surfaces. However, the characteristic of ordering by flow was maintained for only a specific time after filling the channel with the liquid crystalline material. Then, the interaction of the sidewall surfaces was dominant, resulting in the homeotropic alignment fixed on the PDMS channels walls (i.e., with the long axes of the molecules aligned perpendicularly to each channel wall). Such an arrangement of the molecules was maintained in the undisturbed sample. Exemplary images showing the molecular alignment within the channels directly and after some time from filling (for different positioning of the sample concerning the polarizers’ axes) are presented in Figure 7. For an exemplary structure with a straight channel of about 100 μm wide, 30 μm height, and 4 cm length, the homeotropic alignment on the PDMS walls along the entire channel length was observed approximately 10 min after its infiltration with an E7 nematic liquid crystal mixture.

**Figure 8:**
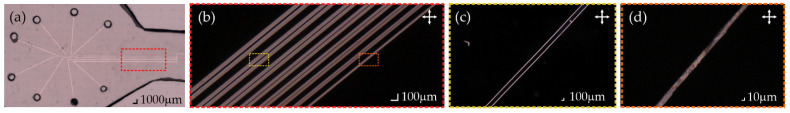
Photo of the test sample consisting of eight channels of different widths from 20 to 250 μm (**a**) and the same structure photographed with the use of a polarizing microscope (with the crossed polarizers) after its filling with a liquid crystalline material (**b**). Two different channels with widths of 200 μm (**c**) and 20 μm (**d**) are shown separately to address the issue of obtaining the homogenous molecular arrangement in the channels with the aspect ratio *w*/*h* < 1. Please note the different magnifications used for images (**c**,**d**). The square boxes in the following panels indicate the regions where the photos were taken.

Since the presence of homeotropic alignment may depend on many factors, including the quality of the structure (resulting, among other things, from the type of mold used for fabrication), type of substrate, geometrical parameters of the structure, or specific liquid crystalline material used, multiple tests on the LC molecular orientation within the channels in the samples of different type were performed. Our previous publication discussed the findings on this issue in detail [19]. To address one important aspect only, it is worth recalling that the structure dimensions are crucial in the successful ordering of LC molecules within LC:PDMS structures and, therefore, should be particularly considered in the design. One of the samples used for testing was a structure consisting of eight channels with widths ranging from 20 to 250 μm (with initial widths increasing by 20 μm intervals and then by 50 μm ones) and a height of 30 μm (see Figure 8a,b). In addition, two channels of different widths (200 and 20 μm) are presented in Figure 8c,d, containing photos taken at different magnifications. It could be observed that the homeotropic anchoring of E7 NLC molecules on PDMS surfaces was much easier to achieve in wider channels. It may even be attempted to define a limiting width below which the liquid crystal molecules do not orient themselves in the system discussed. For more details, please refer to [19], where the LC molecular arrangement in LC:PDMS system was related to the aspect ratio width/height, *w*/*h*). However, additional factors (e.g., quality of the system fabricated in PDMS) should be considered as affecting the actual molecular alignment.

The most crucial matter in the experimental studies was to observe the effect of the external electric field on the LC molecular orientation within LC:PDMS structures. In conducting such investigations, we had two main objectives. The first was to determine if it is possible to obtain a tunable photonic system whose propagation properties could be dynamically controlled by an electric field (keeping in mind that various LC molecular orientations in the structure correspond to different values of the effective refractive index). The second feature was to investigate the effectiveness of using liquid electrodes of various geometries, including those with complex and periodic shapes.

Preliminary studies of the influence of the electric field were performed for the samples of relatively large dimensions, where the width of both the liquid crystal channels and the channels forming the electrodes was about 150–200 μm, and their height was about 30 μm.

**Figure 9:**
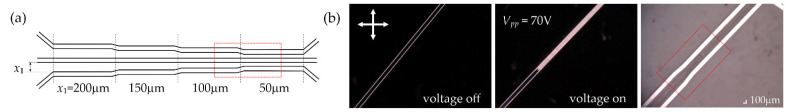
(**a**) Schematic drawing of a structure with gradually approaching electrodes (referred to as Structure 0). The distance of each electrode from the central channel is equal to *x*_1_. (**b**) Experimental results obtained in the LC:PDMS sample of the same geometry with the electric voltage not applied and applied to the electrodes. The third photo was taken with additional (top) illumination to capture the position of the electrodes with respect to the central channel. The square box in both panels indicates corresponding regions in the sample.

Firstly, the structure with symmetric electrodes gradually further away from the central channel (referred to in the rest of this paper as Structure 0) was analyzed to check how the electrode location affects the reorientation efficiency of the LC molecules. The geometry of the system under consideration is shown schematically in Figure 9a, with indications of the distances (*x*) between each side channel and the central one. The starting point for the observations was the homeotropic orientation of the molecules at the PDMS boundary surfaces occurring uniformly along the entire liquid crystal channel with the electric voltage turned off. Subsequently, when the voltage of a given value *V_pp_* was applied to the sample, changes in the molecular orientation were noticed. An example of such observations is presented in Figure 9b as a set of images taken with crossed polarizers before and after the voltage application, as well as a view of the tested structure with additional (top) illumination to illustrate the location of the electrodes with respect to the central channel. From the analysis of the images and based on the observations made, it can be concluded that the application of the electrodes in the form of the microchannels filled with EGaIn gave satisfactory results in the efficient reorientation of LC molecules under the influence of an electric field. However, it can be noticed that for a given value of the voltage (*V_pp_* = 70 V in the presented case), reorientation did not occur along the entire channel length. In fact, the changes in molecular orientation are visible only in the area corresponding to the closest distance of the electrodes from the central channel (it is 50 μm in this case). Therefore, the voltage was gradually increased, making it possible to observe the reorientation effect in the further segments of the structure. Table 1 lists the voltages at which the complete reorientation of the molecules was obtained (i.e., for the long axis of the molecules aligned along the direction of the electric field) for the specific separation (*x*_1_) between each side channel and the central one. Accordingly, the results obtained in this way confirm that the distance of the electrodes from the liquid crystalline channel is a critical factor for the functionality of tunable LC:PDMS devices and should be taken into account when designing the structure geometry.

Another aspect considered in the performed studies was to experimentally verify the possibility of obtaining a periodic optical structure (i.e., with a periodic change in the effective refractive index along the propagation direction). For this purpose, different versions of the structures with periodic electrodes of different geometries were investigated. Based on the results of the numerical simulations, the electrodes were designed to consist of segments that are alternately closer to and further away from the liquid crystal channel.

The values of the geometrical parameters of the structures used in the first part of the experimental tests are summarized in Table 2.

**Figure 10:**
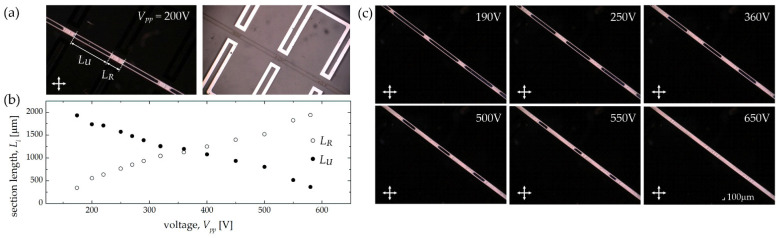
(**a**) Experimental results obtained for Structure 1A when biasing it with an electric voltage of 200 V (peak-to-peak value), showing the periodic rearrangement in the liquid crystal molecular orientation (on the left). The length of the sections corresponding to the reoriented and non-reoriented regions along the central channel are marked as *L_R_* and *L_U_*, respectively. The photo on the right indicates the location of the central channel and periodic electrodes relatively to each other. (**b**) The length of the reoriented (*L_R_*) and non-reoriented (*L_U_*) sections for different values of the applied voltage. The measurement uncertainties were estimated at 20 μm, taking into account the discrepancies between the lengths of the corresponding section on the same image. (**c**) Experimental photos from the polarizing microscope showing the reoriented section’s length increasing with the voltage.

As can be seen, the first two tested structures (Structure 1A and Structure 1B) differed only in the length of the electrode section distant from the central channel, while the other dimensions were the same. The individual microchannels in both cases were 200 μm wide and about 30 μm in height. Starting from the initial state, corresponding to the homeotropic alignment on the channel walls, the LC molecules might be reoriented by applying a sufficiently high electric voltage (the first change in the molecular arrangement was observed for *V_pp_* = 160 V). The result obtained for Structure 1A is presented in Figure 10a, where the periodic rearrangement in the liquid crystal molecular orientation is visible on the left, and the photo of the same structure shown on the right indicates the location of the channel and periodic electrodes. Images were obtained for a peak-to-peak voltage of 200 V, and the reoriented section’s length was about 555 μm (for the length of the electrode section equal to 575 μm) in this case. Although periodic regions of differently ordered LC molecules were thus obtained, it is noteworthy that by increasing the voltage, the range of the changes in LC alignment gradually widened, as illustrated in Figure 10b,c. Eventually, the periodic changes in LC arrangement ceased to be visible at the peak voltages in the order of 650–650 V, for which the influence of the electric field was spatially averaged, and complete reorientation occurred along the entire length of the channel. Such a wide range of driving voltages in the described case (i.e., from 160 to 650 V) results from the specific geometrical parameters of the discussed structure, for which the length of the electrode section closer to the channel (and thus exerting a more substantial influence on the molecular reorientation) was three times shorter than that of one located further away (see Table 2). The level of the reorientation changes was also qualified by measuring the length of both the reoriented and unmodified regions for different values of applied voltages. Specifically, the relevant measures were taken at several analogous locations along the channel, and their average value was calculated. The obtained values are plotted in Figure 10b as a function of the voltage applied.

**Figure 11:**
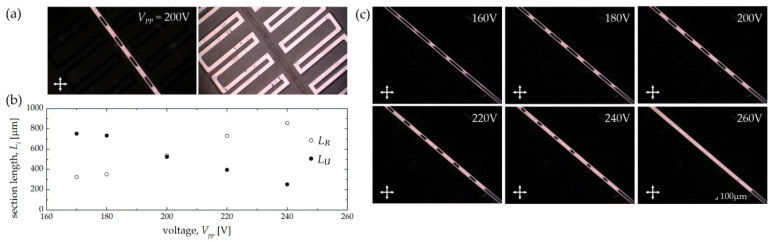
Experimental results obtained in Structure 1B. Descriptions of individual panels (**a**–**c**) are analogous to those used in Figure 10. Please note that a significantly narrower range of the steering voltages was achieved when compared to the one observed for Structure 1A.

While the length of the distant electrode segment was relatively large (*L*_2_ = 1725 μm) in the case of Structure 1A, an analogous electrode geometry but with a two times smaller period (i.e., with *L*_1_ + *L*_2_ = 1150 μm) was also studied. The periodical LC molecular reorientation in the central channel of such a sample (i.e., Structure 1B) when biased with 200 V is shown in Figure 11a. A set of photos illustrating the changes in the molecular alignment when gradually increasing the applied voltage value is presented in Figure 11c. Compared to the first version of the structure, it is worth noting that the reorientation started at a lower peak voltage value (i.e., around 140 V). More importantly, the voltage range for which periodicity in LC molecular arrangement within the central channel was obtained dramatically decreased (i.e., the effect of the electric field was averaged for a voltage value of approximately 260 V). The measured lengths of the reoriented and non-reoriented regions for the specific voltage are summarized in the graph shown in Figure 11b.

The considerations described so far involved structures with relatively large dimensions in terms of the width and height of the individual microchannels and the periodicity of the electrodes. It has to be underlined that their use in the performed research served only as preliminary validations of the assumptions and to obtain some initial conclusions. In fact, such structures cannot be used as waveguides for visible light. Subsequently, experimental works were redirected to miniaturize structures in which the channel width was in the range of 15–50 μm, while their heights were again around 30 μm. When attempting fabrication of the electrodes, some difficulties were experienced in introducing eutectic into the side channels due to the relatively high density of the substance and small dimensions of the entrance inlets. For this reason, it was necessary to find an alternative way to obtain the periodic electrodes by using another conductive material that could be effectively introduced into microchannels of reduced size. Replacing the gallium-indium eutectic alloy with a solution of potassium chloride (3 M KCl). Before this specific electrolyte was applied to the architectures of reduced size, it was necessary to verify the validity of its successful usage in the discussed LC:PDMS systems. For this purpose, the 3 M KCl was introduced into previously examined larger samples (i.e., Structure 0 and Structure 1B, whose microchannels were easily filled with the eutectic) to compare the results obtained for both conducting materials.

**Figure 12:**
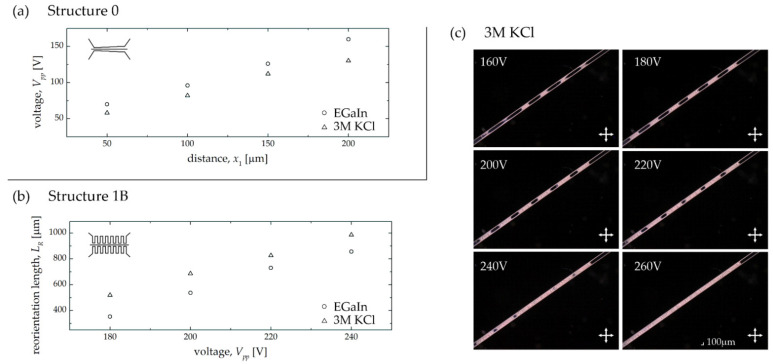
Comparison of the results obtained in Structure 0 (**a**) and Structure 1B (**b**) for the channel electrodes filled with eutectic (EGaIn) and electrolyte (3M KCl). (**c**) Photos from the polarizing microscope showing how LC molecular orientation changes in the central channel when the variable voltage is applied to the side periodic electrodes filled with the 3M KCl solution.

The first sample used for the comparison tests was a system with gradually approaching electrodes (Structure 0, as shown in Figure 9a). During the experimental observations, the peak-to-peak values of the voltage (*V_pp_*) applied to the sample for which the LC molecules were aligned with the electric field direction in the given section of the central channel were recorded. The obtained data for the eutectic and the electrolyte are shown in Figure 12a. An examination of the graph revealed that the values of the voltages for two electrode materials are similar, with the complete reorientation of the liquid crystal molecules occurring for slightly lower voltages when using the electrolyte. Therefore, it can be concluded that using a potassium chloride solution for electrode fabrication in LC:PDMS systems was reasonable and can be successfully implemented in further stages of experimental work.

The electrolyte was then introduced into the structure with periodic electrodes (Structure 1B) to verify whether its application can help to obtain a periodic optical structure consisting of areas with different orientations of liquid crystal molecules. Figure 12c shows exemplary photos of the sample geometry analogous to that analyzed earlier but this time with KCl-based microelectrodes. In addition, the lengths of the reoriented regions for both types of electrode materials are plotted together in Figure 12b. It seems that for the same voltage values, the length of the reoriented region is slightly longer when using the electrolyte, but it may be related to the fact that reorientation occurs at lower voltages for this material (as previously shown in Figure 12a). Nevertheless, it is apparent that the segment lengths of the electrodes are similar, and the dynamic of their widening with increasing voltage is almost the same.

After confirming the suitability of using the 3 M KCl solution for electrodes in LC:PDMS systems, the next step was to perform experiments in the periodic structures of reduced dimensions. It should be emphasized that although the introduction of the electrolyte into the side channels is relatively easy, the possibility of efficient electrical tuning, in this case, is also dependent on the way the LC molecules are initially arranged in the central channel (i.e., before the voltage is applied to the sample). The latter feature appeared to be problematic in the case of narrow channels. For the samples with a central channel characterized by the aspect ratio *w*/*h* < 1, the liquid crystal molecules were typically oriented chaotically without showing any dominant direction in their arrangement after infiltration. Eventually, only two structures (from a comprehensive set of the fabricated samples) with periodic electrodes and with the widths of individual channels of 50 μm and heights of 30 μm were deemed suitable for use at this stage of work.

**Figure 13:**
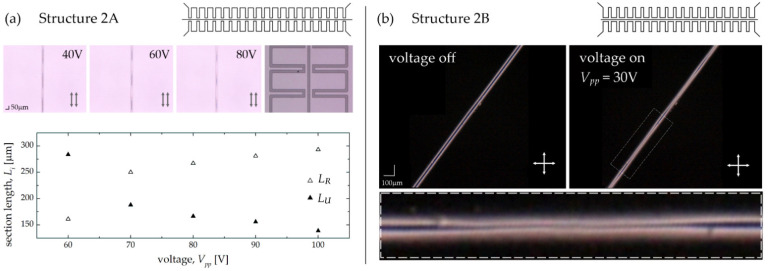
Experimental results obtained in Structure 2A (**a**) and Structure 2B (**b**) when 3 mol/L potassium chloride (3 M KCl) was used to fill periodical electrodes. Observations of the first sample were performed with parallel polarizers’ axes when using POM as the homeotropic alignment of LC molecules was not achieved in the central channel. Only a weak effect of the electric field was reported in the second case.

As shown in Table 3, two versions of the samples were characterized by an inverse sequence of the repeated segments—the lengths of the electrode sections closer and further from the central channel were 150 μm and 300 μm (in Structure 2A), and 300 μm and 150 μm (in the second case—i.e., in Structure 2B), respectively. In the first structure (see Figure 13a for the structure geometry), an axial orientation (i.e., with the long axes of the molecules aligned along the channel axis) was obtained instead of the expected homeotropic one on the microchannel wall surfaces. Nevertheless, we decided to conduct further tests for this structure based on the homogeneous arrangement of LC molecules along the entire channel length. Unlike the observation methodology described so far, the channel axis was set at the angle of 0 degrees to the polarized axis in this case, and the image was observed by setting the polarizer and analyzer axes parallelly to each other. The photos for the selected values of the voltage are presented in Figure 13a. The bright sections visible within the channel refer to the areas where the LC molecules were reoriented (with a long axis aligned along the electric field direction), while the darker ones correspond to the areas where the molecules’ orientation did not change after applying the voltage. As in the previous cases, the correlation between the value of the applied voltage and the length of the reoriented region was investigated, and the obtained results are shown at the bottom of Figure 13a. As also seen for the structure with reduced spatial dimensions, it is possible to electrically control the periodicity of effective refractive index changes along the liquid crystalline channel. Moreover, the values of the voltages needed to induce molecular reorientation are lower than in the case of structures with corresponding geometry but larger dimensions.

For the second sample (Structure 2B), it was possible to obtain a homeotropic anchoring of the LC molecules on channel walls. For this reason, the latter was positioned at an angle of 45 degrees with respect to the crossed polarizers’ axes during the experimental observations. Photos of the structure taken without applied voltage and when biasing the sample with *V_pp_* = 30 V are shown in the central part of Figure 13b. The length of the reoriented region in the presented case was about 285 μm for a length of the electrode segment located in the vicinity of the channel equal to 300 μm (please see the close up of the photo taken with the voltage applied). Notably, the voltage required to induce reorientation over such a long segment is noticeably lower when compared to all cases discussed in this report. It is related to the closest distance of the electrode from the central channel (for both segments) and the shorter length of the further fragment (compared to the previously described sample referred to as Structure 2A).

Considering the experimental results obtained, further downsizing of the structures is reasonable (especially when considering practical applications in optofluidic waveguiding structures), but unavoidably combined with a decrease in the height of the channels (to obtain suitable ordering of LC molecules within a waveguiding channel). Preliminary experimental studies for structures with channels of single micrometer height were carried out, and the results obtained will be presented elsewhere. It is worth noting that the main research direction in further redesigning the sample to optimize its performance is bringing the electrodes closer to the central channel, which would effectively reduce the driving voltage value. Keeping in mind that minimizing the periodicity of the meander electrodes (which are required to get the optical structure in the Bragg grating type) is essential in the subsequent samples, we are considering elevating the height of the electrode channels (which could additionally simplify their infiltration with the metal eutectic). Obtaining the shorter periods of the spatial effective refractive index modulation within the liquid crystalline channel may lead to the creation of the tunable Bragg reflector or electro-switchable diffractive grating analogous to those described in [15] and [37], respectively. Moreover, we consider the LC:PDMS structures for possible application as long-period gratings (LPG). For this purpose, complex numerical studies have to be performed, including the more accurate determination of the LC molecular reorientation under a periodic electric field, as well as the implementation of a suitable model for LPG description (e.g., the coupled-mode theory) [81,82,83]. Due to the limited volume of the manuscript, detailed results on this subject will be reported in a future publication.

The stability of LC:PDMS devices was monitored for a time period of a few months, with no deterioration in their switching properties or changes in their electro-optical performance observed. The most crucial aspect to be considered when studying the cyclical stability of the presented device is the stability of the LC molecular alignment obtained in the LC:PDMS microstructures, and it was proven to not vary with time (as mentioned in our previous paper [19]). Moreover, it is worth underlining that the studies of the other research groups have not indicated any instabilities when it comes to repeatable driving PDMS-based VA LC cells with the electric field, with no issues related to electro-optical performance maintenance [22].

## 4. Conclusions

The paper discusses the design, fabrication, and testing methods for electrically tuned LC:PDMS structures. It represents an innovative solution related to the use of microchannel electrodes filled with conductive liquids. The latter allows for the easy and dynamic reconfiguration of the system and eliminates technological issues experienced by other research teams using typical metallic electrodes sputtered directly onto the PDMS surface. Moreover, the recommended method of electrodes fabrication in LC:PDMS structures is a relatively fast and unsophisticated technique, as it does not require additional technological processes to adapt the samples for proper and efficient operation. The presented studies provide a general idea and the first set of geometrical parameters that need to be checked in the experimental conditions, although a quantitative comparison between the numerical and experimental data cannot be easily realized due to the significant approximations made when performing the calculations. The results of numerical simulations and experimental tests demonstrate that the position of the electrode with respect to the central channel is an essential parameter that should be carefully adjusted when designing the structure. Based on this fact, various configurations of the electrodes have been proposed, including electrodes with periodically varying distances, making it possible to obtain periodic changes in the spatial distribution of the electric field intensity within the liquid crystal channel. The optimal geometrical parameters of the periodic electrodes (in the sense of obtaining maximal modulation of the electric field intensity in space) were selected based on a series of numerical simulations. The obtained results show that by designing the electrode geometry appropriately, the optical properties of the LC:PDMS optofluidic structures can be easily controlled and steered. It is worth noting that there is a large number of possible geometrical configurations that can be freely changed and adapted to the planned applications. The alternatives considered and presented in this paper are options that provide the basis for further developing this type of optical system based on electrically controlled LC:PDMS structures.

Thanks to the proposed approach related to electrodes fabrication, the LC:PDMS structures may be easily tunable and controllable using an electric field. Their potential applications include low-power photonic components for integrated optics (lab-on-chip) and waveguiding systems. Moreover, they can be used for the sensing and monitoring of other external fields and factors.

## Figures and Tables

**Table 1:** The voltages required to align LC molecules along the electric field direction for different distances of the electrodes from the central microchannel with liquid crystalline material.

**Electrode Distance *x*_1_ [μm]**	50	100	150	200
**Peak-to-Peak Voltage Applied *V_pp_* [V]**	70	96	126	160

**Table 2:** Geometrical parameters of the periodic electrodes in the structures used for the initial tests in experimental conditions.

	*x*_1_ [μm]	*L*_1_ [μm]	*x*_2_ [μm]	*L*_2_ [μm]
Structure 1A	200	575	2780	1725
Structure 1B	200	575	2780	575

**Table 3:** Geometrical parameters of the periodic electrodes in the second batch of samples tested in experimental conditions.

	*x*_1_ [μm]	*L*_1_ [μm]	*x*_2_ [μm]	*L*_2_ [μm]
Structure 2A	50	150	705	300
Structure 2B	50	300	695	150
